# Correction: ADHD and family life: A cross-sectional study of ADHD prevalence among pupils in China and factors associated with parental depression

**DOI:** 10.1371/journal.pone.0326427

**Published:** 2025-06-13

**Authors:** Tao Lv, Longlong Li, Ying Tang, Gerard Leavey

The first author’s name is spelled incorrectly. The correct name is: Tao Lv. The correct citation is: Lv T, Li L, Tang Y, Leavey G (2024) ADHD and family life: A cross-sectional study of ADHD prevalence among pupils in China and factors associated with parental depression. PLoS ONE 19(3): e0281226. https://doi.org/10.1371/journal.pone.0281226.
